# Seroprevalence and genotypic characterization of HBV among low risk voluntary blood donors in Nairobi, Kenya

**DOI:** 10.1186/s12985-020-01447-2

**Published:** 2020-11-13

**Authors:** Patrick Okoti Aluora, Margaret Wangui Muturi, George Gachara

**Affiliations:** 1 Department of Health and Applied Sciences, Nairobi Technical Training Institute, Nairobi, Kenya; 2 Department of Medical Laboratory Science, Kenyatta University, Nairobi, Kenya

**Keywords:** Occult HBV infection, Hepatitis, Liver cirrhosis, Hepatocellular carcinoma, Mutations, Escape mutations, Undetectable

## Abstract

**Background:**

Hepatitis B virus (HBV) causes significant morbidity and mortality globally primarily due to its ability to cause hepatitis, liver cirrhosis and hepatocellular carcinoma. The Kenya National Blood Transfusion Services screens for Hepatitis B antibodies using the chemiluminescent microparticle immunoassay method. This test does not inform on the genotypic characteristics of the virus or the actual presence of the virus in blood. This study therefore sought to determine the serologic and genotypic profiles of HBV circulating among the voluntary blood donors in Nairobi.

**Methods:**

Blood samples were collected in plain and EDTA vacutainers and tested for the Hepatitis B surface antigen (HBsAg). HBV DNA was then extracted from plasma, its overlapping P/S gene amplified and sequenced. The resulting sequences were used to analyze for the circulating genotypes and mutations within the P and S genes. Bivariate statistical analysis was used to determine the association between demographic factors and HBV infection.

**Results:**

A seroprevalence of 2.3% (n = 7) was reported. The age group 19–28 years was significantly associated with HBV infection. Nine samples were positive for HBV DNA; these included 2 HBsAg positive samples and 7 HBsAg negative samples. Genotype A, sub genotype A1 was found to be exclusively prevalent while a number of mutations were reported in the “a” determinant segment of the major hydrophilic region of the S gene associated with antibody escape. RT mutations including mutation rt181T in the P gene conferring resistance against Lamivudine and other ʟ-nucleoside drugs were detected.

**Conclusion:**

There is a high prevalence of occult HBV infections among these blood donors and therefore the testing platform currently in use requires revision.

## Background

Globally, about 2 billion people are infected with Hepatitis B virus (HBV), with about 360 million at risk of developing complications due to the infection. Of the 2 billion infected individuals, more than 65 million residing in Africa are chronically infected and are therefore at risk of HBV related complications. These complications include liver cirrhosis and hepatocellular carcinoma. Sub Saharan Africa records the highest HBV prevalence in Africa with prevalence varying from one country to another [1–3]. In Kenya, studies among different risk groups report prevalence between 5 and 30% [4]. The studied groups in Kenya so far have included drug users, pregnant women, children attending post natal clinics, people living with HIV/AIDS, blood donors and patients in specific regions around the country.

HBV is mainly transmitted through direct exposure to infected blood or other body fluids. Consequently, pre-transfusion screening of blood for HBV and other transfusion transmissible infections (TTIs) to avoid the risk of iatrogenic transmission has become standard procedure. The TTIs currently screened in Kenya besides HBV are Human immune-deficiency virus (HIV), Syphilis, Hepatitis C virus (HCV) and depending with the geographical area, some regions screen for malaria. The current screening for HBV in donor blood in Kenya involves serological detection of the Hepatitis B surface antigen (HBsAg) which is a marker of active infection using the chemiluminescent microparticle immunoassay (CMIA). This testing does not provide the genotypic characteristics after diagnosis hence letting those diagnosed pursue this privately, if they wish to do so. Additionally, a negative test result with this and other serological methods does not completely exclude the actual presence of the virus.

HBV infection is characterized by waxing and waning trends where it goes through an alternating replicative and non-replicative cycles. People infected with HBV can develop acute symptoms and attain a complete immune clearance of the virus thus yielding a lifelong immunity [5]. On the other hand, the alternate fate of the infected people is the development of a chronic status. The three major stages of infection include immune tolerant phase, immune clearance and inactive carrier phase. The inactive carrier state may occur with either alternating reactivations or not.

Infected individuals remain Hepatitis B e-antigen (HBeAg) positive with high concentrations of HBV DNA in their blood. HBeAg is a secreted protein expressed by every member of *Hepadnaviridae* family though its expression is not required to maintain infection [6]. In some infected individuals, symptoms may not develop or even experience minimal histological activities in the liver. The immune tolerance phase is the most infective stage and it lasts for about 2–4 weeks. The last stage involves the immune clearance phase and may last for months or years before one gets to the carrier phase. The carrier stage is characterized by the seroconversion of HBeAg to HBeAb and the HBV DNA may become non-detectable [5, 7, 8].

The HBV genome is a relaxed circular DNA (rcDNA) that is partially double stranded. The genome comprises of 4 overlapping Open reading frames (ORFs) each translated into different components of the virus structure. The overlapping structure of the coding regions facilitates the use of HBV genome with high efficiency during replication [9]. HBV is currently categorized into ten different genotypes, A-J based on more than 8% nucleotide divergence that exists in the HBV genome [10–12]. Two of the genotypes (A and D) are further classified into sub genotypes. This is based on 4–8% intergroup nucleotide difference across the complete genome with good bootstrap support [13, 14]. Studies have shown that the different genotypes and sub genotypes show distinct geographical distributions. For example, three of the ten genotypes, A, D, E, are more prevalent in Africa while Genotype C has been described in some African populations though less prevalent compared to the other three.

In Kenya, genotypes A, D, and E have been reported with genotype A being dominant in most studied populations. According to Webale et al. [15], HBV genotype A, sub genotype A1 was found to have a high prevalence among HIV-1 infected adults. These findings were similar to previous studies that had been conducted prior to 2015. A previous study among voluntary blood donors across the country identified genotype A, sub genotype A1 and genotype D, sub genotype D4 as the most prevalent [4]. Since the genetic diversity of viruses shows spatio-temporal variations, this study sought to determine the circulating HBV genotypes among voluntary blood donors in Nairobi, Kenya.

## Methods

### Study setting

The study was conducted among voluntary blood donors at the Nairobi regional blood transfusion centre (NrBTC). The center offers blood collection from voluntary and substitution blood donors, screening and processing it for different blood products. Screening for HBV at this centre follows the national guidelines which involve detection of HBsAg using the CMIA as the primary method and the enzyme linked immunosorbent assay (ELISA) as a backup. The tested blood and processed products are then distributed to different hospitals for transfusion to patients.

### Study populations and ethical considerations

This was a cross-sectional study conducted among voluntary blood donors who met the criteria for blood donation as per the national guidelines. The study was approved by the Kenyatta University Ethics and Review Committee (KU-ERC). Voluntary blood donors were not coerced nor remunerated to take part in the study.

#### Inclusion criteria

All donors who met the donation requirements as provided by the Kenya National Blood Transfusion Services (KNBTS) for donation, qualified for inclusion into the study. These requirements included;

- The donor had to be aged between 16 and 65 years for donation though for inclusion in the study one had to be aged between 18 and 65 years.
- The donor had to have a body weight of not less than 50 kg.
- The individual’s haemoglobin of not less than 12.5 g/dl and informed written consent to participate in the study.

#### Exclusion criteria

People who did not qualify for blood donation as provided for in the KNBTS questionnaire were excluded from the study. These included;

- Individuals younger than 16 years and older than 65 years and for this study individual younger than 18 years and older than 65 years were not allowed to participate.
- Individuals with body weight less than 50 kg.
- Individuals with haemoglobin less than 12.5 g/dl and those who did not sign consent to participate in the study.

### Sample size determination

The minimum sample size (n = 178) was calculated using the modified Fischer’s formula [16] utilizing a 13.3% HBV seroprevalence among blood donors in Nairobi according to Kerubo et al. [1]. However, a larger sample size (n = 300) was collected and analyzed to allow for an adequate statistical power with 10–15 samples collected per day.

### Sample collection and processing

The participants were randomly selected from the different blood donation sites as they were determined by the NrBTS from time to time. The study protocol was explained to them and upon giving written consent; they were given questionnaires seeking information about their demographic characteristics. After blood donation, 5mls was tapped immediately into a plain vacutainer from the donor’s blood bag extension tube followed by another 5mls into an EDTA vacutainer. The samples were then coded for confidentiality. The collected plain tube and EDTA vacutainer samples were used to obtain serum and plasma respectively after centrifugation at 1500 rpm for 10 min. Serum and plasma obtained were then aliquoted into cryovials and stored at − 80 °C awaiting further processing.

### HBV serology

Sera from all the samples were screened for HBV infection by the chemiluminescent microparticle immunoassay (CMIA) using the Architect i2000 SR system according to the manufacturer’s instructions.

### DNA extraction

The HBV DNA was extracted from 200 µl of all the collected plasma samples using the *QIAGEN* DNeasy DNA extraction kit (*QIAGen,* Hilden, Germany) according to the manufacturer’s instructions.

### PCR and sequencing

Amplification of the overlapping HBV P and S genes was carried out on all samples using a nested PCR protocol described previously [17]. The first round reaction was performed in a 25 µl DreamTaq PCR master mix (Thermo Fisher Scientific Inc., USA) containing DreamTaq DNA polymerase, optimized DreamTaq buffer, MgCl_2_ and dNTPs. The samples were subjected to an initial denaturation at 94 °C for 2 min followed by 35 cycles involving denaturation at 94 °C for 45 s, annealing at 53 °C for 30 s, and extension at 72 °C for 30 s. Then a final extension at 72 °C for 2 min. Five µl of the first round PCR product was used as a template for the nested PCR under the same reaction conditions but with inner primers. Positive and negative controls were used in each run. The PCR products were then resolved by a 1.5% agarose gel electrophoresis and interpreted under UV light. HBV PCR positive amplicons were then purified by treating with shrimp alkaline phosphatase exonuclease I (ExoSapI) and sequenced directly using the Sanger method on a 3500 XL Genetic Analyzer (Applied Biosystems, CA, USA).

### Data analysis

All the collected demographic data (gender, level of education and age) was tabulated and entered in a Microsoft Excel® spreadsheet and then exported for analysis into SPSS version 20 (IBM, Chicago, IL, USA). The seroprevalence of HBV among the donors was determined as the proportion of those individuals testing positive by CMIA. Bivariate statistical analysis was used to determine the association between demographic factors and HBV infection. The variables were considered associated with HBV infection with a *p* value of < 0.05.

The resulting HBV contiguous nucleotide sequences of the study samples were manually assembled and edited using BioEdit ver 7.2.5 [18] and then aligned with a reference sequence (gene bank accession number KP168426.1) using ClustalW implemented in MEGA X [19]. A phylogenetic tree was then constructed with the MEGA X software using the maximum likelihood method [20]. The robustness of the tree was assessed by 1000 bootstrap replicates [21]. The HBV sequences obtained were also analyzed to determine their genotypes and to assess possible drug resistance and immune escape mutations using two online tools Geno2Pheno HBV database (HBVdb) (https://hbv.geno2pheno.org/) and HBVseq program from HIV Stanford database (https://hivdb.stanford.edu/HBV/HBVseq/development/HBVseq.html). The resulting sequences were deposited in the gene bank under accession numbers MT185642-MT185650.

## Results

### Study population

There was a total of 300 voluntary blood donors who were recruited in the study majority of whom were male; 59.3% (*n* = 178) while 0.7% (*n* = 2) were not willing to disclose their gender. Majority of the study participants had a tertiary level of education at 69% (*n* = 207), 18% (*n* = 54) had a secondary school education, 2.3% (*n* = 7) had primary level of education while 10.7% (*n* = 32) did not disclose their level of education. Majority of the study participants, 50.67% (*n* = 152) were aged between 19 and 28 years, 33.3% (*n* = 100) were aged between 29 and 38 years, while 11% (*n* = 33) were aged between 39 and 48 years. Of the remainder, 2.6% (*n* = *8*) were aged between 49 and 58 years, 1.0% (*n* = *3*) were aged between 59 and 68 years and 1.3% (*n* = *4*) were not willing to declare their age but were willing to participate in the study.

### Serology

Based on the chemiluminescent microparticle immunoassay (CMIA) results, 97.6% (*n* = 293) samples tested HBsAg negative while 2.3% (*n* = 7) tested positive. These results, gives an HBV seroprevalence of 2.3% among blood donors in Nairobi.

### Demographic factors and their association with HBV infection

Education was not associated with HBV sero-positivity among the blood donors (*p* = 0.993) neither was there an association between HBV sero-status and gender (*p* = 0.658). There was however a significant association between HBV sero-positivity and age. Participants aged between 19 and 28 years were more likely to be HBsAg positive (*p* = 0.001) compared to the other age groups.

### Prevalent HBV genotypes

**Fig. 1:**
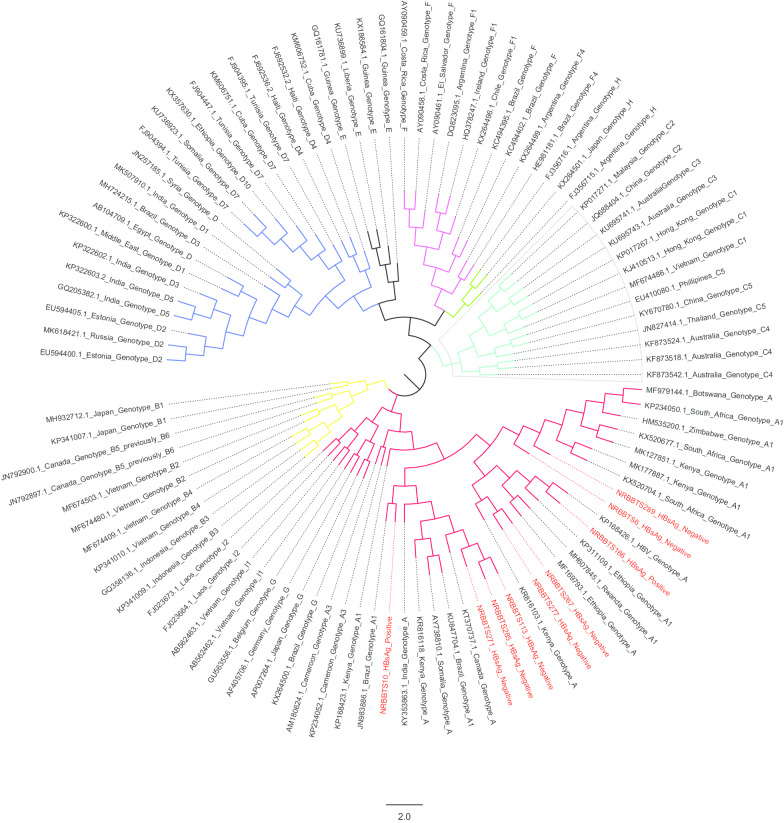
Unrooted phylogenetic tree of HBV sequences from this study and selected global sequences. The nine sequences from this study are written in red with their HBsAg status indicated next to them and fall in genotype A, Sub-genotype A1. All reference sequences are labeled with their gene bank accession numbers, sub genotypes and country of origin

HBV DNA amplification was successful in only 2 of the 7 HBsAg positive samples and was possible in 7 of the 293 HBsAg negative samples hence giving an occult HBV infection (OBI) prevalence of 2.4% (7/293). Of the 9 HBV DNA successfully amplified samples, 3 were from male donors while 6 were from female donors. Phylogenetic analysis revealed that all the viruses belonged to genotype A as shown in Fig. 1. Further analysis showed that, they belonged to sub genotype A1 and were 96–100% similar with an average identity of 96.48% at the nucleotide level (Table 1).

**Table 1:** Demographic factors and their association to HBV infection

Demographic factor	HBsAg status		*p* value
	Positive	Negative	
Gender			0.993
Male	3	175	
Female	4	116	
Undeclared gender	0	2	
Level of education			0.658
Primary	0	7	
Secondary	1	53	
Tertiary	5	202	
Undeclared	1	31	
Age (years)			0.001
19–28	4	148	
29–38	1	99	
39–48	2	31	
49–58	0	8	
59–68	0	3	
Undeclared	0	4	

These are the frequencies of each demographic value in relation to HBsAg status of the study participants. The table also includes the *p *values for each of the demographic factors identified in relation to HBsAg status

### HBsAg escape mutations

Escape mutations in the S gene’s major hydrophilic region (MHR) as well as outside the MHR are shown in Table 2. These mutations are associated with poor detection in serological assays. A total of 6 mutations were described in the S region. Three of these mutations were located within the “a” region of the s gene i.e. T143M, M133T, D144G, while 3 were located within the MHR but outside the “a” region i.e. T114P, A159V, F158L. Three mutations were noted prevalent in the S region but outside the MHR, i.e. Y206E, A194V, S207K.

**Table 2:** S gene mutations from the current study

HBV S gene region	Mutation	Accession number	HBsAg Sero status
MHR (aa 99–169)			
‘a’ determinant region (aa 124–147)	T143M	MT185642	Negative
		MT185646	Negative
	M133T	MT185649	
	D144G	MT185649	Positive
Outside ‘a’ determinant region (aa 99–123 and 148–169)	T114P	MT185645	Negative
		MT185643	Negative
		MT185647	Negative
	A159V	MT185642	Negative
		MT185646	Negative
	F158L	MT185643	Negative
Downstream of the MHR (aa 170–207)	A194V	MT185650	Negative
		MT185649	Positive
		MT185648	Negative
	Y206E	MT185642	Negative
		MT185646	Negative
	S207K	MT185650	Negative
		MT185649	Positive

These mutations were identified using two online tools Geno2Pheno HBV database (HBVdb) (https://hbv.geno2pheno.org/) and HBVseq program from HIV Stanford database (https://hivdb.stanford.edu/HBV/HBVseq/development/HBVseq.html). The mutations are listed for each of the sample where they occurred

### Drug resistant mutations

HBV P gene mutations associated with drug resistance were described. Drug resistance mutations were detected in several samples. Mutation rt181T conferring resistance against Lamivudine (Zeffix®), Adefovir (Hepsera®) and Telbivudine (Tyzeka® & Sebivo®) was described in one sample. M129L and V163I mutations conferring resistance against lamivudine were the most prevalent in the RT gene described in all the 9 samples. The other mutations described in the RT region were S202G, N122P, Y126H, S109P, N122H, S202R, S202P, and M204R (Table 3).

**Table 3:** Drug resistance conferring mutations from the current study

Accession number	Mutations	Drug resisted	HBsAg sero status
MT185643	Y126H	Adefovir dipivoxil(ADV)	Negative
	M129L	Lamivudine	
	V163I	Lamivudine	
MT185642	Y126H	Adefovir dipivoxil(ADV)	Negative
	M129L	Lamivudine	
	V163I	Lamivudine	
MT185644	Y126H	Adefovir dipivoxil(ADV)	Positive
	M129L	Lamivudine	
	V163I	Lamivudine	
MT185645	Y126H	Adefovir dipivoxil(ADV)	Negative
	M129L	Lamivudine	
	V163I	Lamivudine	
	S202G	Entecavir & Lamivudine	
MT185646	Y126H	Adefovir dipivoxil(ADV)	Negative
	M129L	Lamivudine	
	V163I	Lamivudine	
MT185647	Y126H	Adefovir dipivoxil(ADV)	Negative
	M129L	Lamivudine	
	V163I	Lamivudine	
MT185648	M129L	Lamivudine	Negative
	V163I	Lamivudine	
MT185649	M129L	Lamivudine	Positive
	V163I	Lamivudine	
MT185650	M129L	Lamivudine	Negative
	V163I,	Lamivudine	
	A181T	Lamivudine (zeffix), Adefovir(Hepsera), Telbivudine(Tyzeka and Sebivo)	

These mutations were identified using two online tools Geno2Pheno HBV database (HBVdb) (https://hbv.geno2pheno.org/) and HBVseq program from HIV Stanford database (https://hivdb.stanford.edu/HBV/HBVseq/development/HBVseq.html). The mutations are listed for each of the sample where they occurred

## Discussion

The current study has demonstrated a 2.3% seroprevalence of HBV among blood donors in Nairobi. This prevalence is lower compared to a 5.6% seroprevalence reported among the same group of people in Nakuru and Tenwek Mission hospital, Kenya, using ELISA testing [22] and similar to 2.4% reported among blood donors in Nyeri in 2016 on samples collected in 2014 using Murex HBsAg version 3 which is a rapid and sensitive enzyme immunoassay [23]. On other hand, this prevalence is however higher than the 0.4% reported in Eldoret in 2018 [24] and lower than the 3.46% described in Kisumu, Homabay and Siaya counties in 2018 [25]. These differences in seroprevalence could be attributed to variations in geographical locations and the sensitivity of the screening techniques used in the detection of the HBsAg for the different studies.

There was a significantly higher prevalence of HBsAg among donors aged 28 years and below contrary to the expectation that it could have been lower since the HBV vaccine was introduced in the country in 2001 [26]. This finding is however different from those of recent studies in Kenya which recorded a higher HBV prevalence in higher age groups. Kiyeng et al. [24] found that HBV prevalence was higher in age group 31 years to 40 years and that there was a significant increase in positivity with increase in age. In their study, Bartonjo et al. [22] recorded that a majority of donors in Tenwek with TTIs were aged between 36 and 40 years. This finding may be attributed to mutations in the B cell epitopes (aa106–aa117, aa122–aa148, and aa160–aa207) and T cell epitopes (aa94–aa105, aa106–aa117, and aa136–aa155) in the S gene of the HBV genome. Mutations in these epitope-regions could change their humoral and cellular immunity epitopes of Pre-S, S and polymerase and lead to HBV infection even in the vaccinated population [27]. Prevalent B-cell and T-cell epitopes mutations detected in this study include S109P, T114P, N122P, N122H, Y126H, M129L, M133T and T143M. These mutations could have developed in the study subjects or, they could have been infected with immune escape mutants.

The finding of this study that the prevalent genotype among the blood donors is A sub genotype A1, agrees with findings from a similar study reported by Owuor et al. [4]. This current study did not identify any other genotype although, other studies have found genotypes D [4, 28] and E [29]. The dominance of genotype A locally has been previously confirmed by other studies conducted among different groups in Kenya [15, 28, 30, 31].

Studies conducted in the recent past show that the sensitivity of serological assays is primarily affected by mutations in the HBsAg portion of the S gene. These mutations occur in the “a” determinant region (aa124–aa147) in the major hydrophilic region (MHR) that spun from aa99–aa169 which can cause false negatives when HBsAg alone is used in HBV screening. In this study, three mutations were identified in the “a” determinant region of the s gene. T143M was found in two different samples with M133T and D144G occurring in one sample as well. Of the three mutations, T143M affected the detection of the HBsAg in the samples since this mutation was only detected in HBsAg negative samples. This finding is similar to a previous study reported by Nyairo et al. [33]. T143M mutation is a known mutation that has been previously described to alter antigenic properties of variant HBsAg. It has also been associated with problems in diagnostic assays and escape to vaccine and HBIg therapy [32, 33].

Two mutations occurring downstream of the MHR namely S207K, A194V and other two within the MHR region but outside the “a” determinant region; T114P, F158L, as well affected the detection of the HBsAg since the antigen was not detected in the samples with these mutations. These findings agree with previous studies that found out that mutations within the “a” determinant region as well as other regions of the S gene can affect the diagnosis and detection of vaccine-escaped Hepatitis B infection [34]. The extraction of HBV DNA from HBsAg negative samples in this population can therefore be attributed to the circulation of viruses with mutations that interfere with HBsAg recognition by antibodies in serological assays. Two escape mutations were also detected in one sample, M133T and D144G, associated with detection and vaccine/therapy, respectively. There was however no information on HBV vaccination or treatment history of the specific donors with these mutations.

HBV DNA was extracted in 7 samples of the HBsAg negative samples giving an OBI prevalence of 2.3%. This study hence confirms the presence of OBI in this population. This can be attributed to the mutations described in the S gene. Generally, all the seven OBI positive samples possessed mutations within the MHR region of the S gene and also the region downstream of the MHR compared to only one of the HBsAg positive samples. These samples were declared negative and the blood released for transfusion while they were actually positive for HBV infection. Though the prevalence reported here is higher compared to previous findings by Langat et al. who reported no occult infection among the blood donors, this study agrees with previous study by Mabeya et. al. conducted later on who reported a prevalence of 2.25% of OBI among HIV patients [35, 36]. The prevalence is however low compared to a prevalence of between 8.3% to 30.8% reported by Jepkemei et al. [37] among different high risk groups of HBV infection [37].

Majority of the mutations in the P gene described in this study were primary drug resistance mutations that decrease susceptibility to nucleoside analogues. Among the sequences, one had a mutation A181T conferring resistance to Lamivudine (LMV). This mutation affects the HBV reverse transcriptase enzymatic activity required for viral replication leading to potential drug resistance and progression of the liver disease [38]. LMV is widely used in Kenya for HIV treatment as well as in pre and post exposure prophylaxis. Though it was not determined if this patient had exposure to the drug and other nucleoside analogues in the past, rt181 mutation has been widely described in antiretroviral-naïve individuals among different cohorts worldwide. M129L and V163I mutations were the most prevalent in the RT gene. These mutations have been described previously in both treatment naïve and LMV experienced patients with a strong association with genotype A [39, 40]. Several mutations identified in this study have previously been described to cause resistance against specific drugs. As Choi et al. [38] records, mutation Y126H detected in this study has been previously described to cause resistance against Adefovir dipivoxil (ADV), while S202G mutation conferring resistance against Entecavir and Lamivudine was also described. Mutation Q215E has as well previously been described to cause resistance against LMV and ADV [38].

### Study limitations

This study did not look into data on HBV viral loads, more serological markers and the liver enzymes particularly of the affected individuals. This hence precludes the ability to correlate the study findings and the clinical outcomes of the affected patients. The study as well did not conduct a follow up on the individuals to monitor their progression after diagnosis hence no finding was reported herein on the pattern of disease progression particularly in individuals with occult infections.

## Conclusions

In conclusion, the findings of our study confirms the circulation of genotype A in Kenya and identifies occult HBV infections not previously reported in this population. These study findings necessitates the need to review the national protocol for screening of HBV to ensure safety of blood and blood products given to patients. Additionally, the study reaffirms the need to incorporate nucleic acid tests in HBV screening.

## Data Availability

All data generated or analyzed during this study are included in this published article and its supplementary information files. The sequences were deposited in the gene bank under accession numbers MT185642-MT185650.

## Abbreviations

AIDS: Acquired immune deficiency syndrome
CMIA: Chemiluminescent microparticle immunoassay
DNA: Deoxyribonucleic acid
EDTA: Ethylenediamine tetra-acetic acid
ELISA: Enzyme-linked immunosorbent assay
HBV: Hepatitis B virus
HBsAg: Hepatitis B surface antigen
HCC: Hepatocellular carcinoma
HIV: Human immunodeficiency virus
KNBTS: Kenya National Blood Transfusion Services
ORF: Open reading frame
PCR: Polymerase chain reaction
rcDNA: Relaxed circular deoxyribonucleic acid
SPSS: Statistical package for the social Sciences
